# Analysis of psychic imbalance, caused by screening of a video of surgical extraction of a lower third molar in a sample of mental patients as compared to the general population

**DOI:** 10.4317/jced.59861

**Published:** 2022-09-01

**Authors:** Elena Bermúdez-Bejarano, Juan-Antonio Bermúdez-Sánchez, Francisco-José Ruiz-Rey, María-Ángeles Serrera-Figallo, José-Luis Gutiérrez-Pérez, Daniel Torres-Lagares

**Affiliations:** 1 Master’s in Advanced Oral Surgery. University of Seville; 2 PhD in Medicine and Surgery, psychiatrist and forensic psychiatry. Malaga; 3 PhD in Education and Expert in Statistics. University of Malaga; 4 Lecturer, Department of Stomatology. University of Seville; 5 Full Professor, Master’s in Oral Surgery. University of Seville

## Abstract

**Background:**

The goal of this study is to validate the psychometric properties of the Hamilton Rating Scales for anxiety and depression. These two scales will be used to analyze anxiety and depression, seven days before, after and seven days after screening of a video showing ex-traction of a lower third molar in four different strata of the sample: mixed disorder, anxiety dis-order, adaptive disorder, and no mental disorder.

**Material and Methods:**

A prospective study was performed of 240 Caucasian subjects ages 18-70 in a psychiatry outpatient clinic in Malaga. The study was ap-proved by the Research Ethics Committee of the University of Seville. Following interviews with a psychiatrist and completion of the Hamilton scales, the participants were divided into four levels, with 60 participants per group. The influence of sex and place of residence were analysed.

**Results:**

The scales showed good psychometric properties. At the three video screenings, the means were higher for women, persons from rural environments and persons with mixed disorder in the first instance and then anxiety disorder.

**Conclusions:**

Patients with mixed disorder experience a higher level of anxiety and depression than do patients free of mental pathologies.

** Key words:**Anxiety disorder, adaptive disorder, dental anxiety, mixed anxiety-depressive disorder, surgical extraction.

## Introduction

The general population shows a marked level of fear, anxiety, phobia and even rejection of everything to do with dental treatment. These circumstances are accentuated when the therapy requires treatment with oral surgery. The Diagnostic and Statistical Manual of Mental Disorders distinguishes between fear as a warning signal—response to a nonconflictive, definite, external, known influence that prepares the organism to defend itself—and the different and opposing concept of anxiety, replica of a conflictive, vague, internal or unknown threat that blocks the subject that suffers from it (1). Dental fear and dental anxiety belong to the sphere of anxiety, and both have a strong influence on oral and public health (2-4).

A higher degree of general anxiety is directly proportional to a higher level of dental anxiety and dental fear, each of which is its own psychological disorder (5). The latter are associated with a significant need for dental treatment, which in turn fosters more invasive and less restorative therapies (6). This phenomenon leads to fewer visits to the dentist due to avoidance or postponement, contributing to a vicious cycle (2,3,7-9) and decline in oral health (7,8,10).

One of the most common procedures in oral surgery is tooth extraction, causing anxiety and an unpleasant feeling, being intensified if it is an impacted third molar and in need of surgical exodontia (11). A direct correlation exists between degree of surgical stress and the procedure itself (12), and between the intensity of pain perceived and the level of dental fear and/or dental anxiety (13,14). Providing pre-operatory information to control the patient’s anxiety can be counterproductive, since it can (15) cause anxiety to peak after watching a video with the necessary information on the therapy to be performed (16-18). Some studies extol giving patients such information (19,20), however, arguing that there are two types of patient. These studies argue that the intervention has positive results for so-called “vigilant” patients, who attempt to overcome stressful situations by obtaining the most information possible, whereas “evasive” patients may reject any type of information (21).

This study focuses on three disorders. Adaptive disorders involve a series of symptoms including episodes of sadness, emptiness, lack of interest, involuntary weight change, insomnia and/or hypersomnia, agitation, psychomotor delay, energy loss, low self-esteem, indecision, decreased capacity, and recurring thoughts of death and/or suicide (1). In anxiety disorders, anxiety can occur with any eventuality that threatens identity and/or aggression to the self. If anxiety becomes too intense, frequent or persistent and interferes with daily life, however, it can become part of an anxiety disorder (22). Finally, mixed anxiety-depressive disorders are psychological profiles that present symptoms of both associated disorders but in which neither disorder is predominant and thus does not justify separate diagnosis (23).

All of these mental disorders are pathologies that not only involve deterioration in the psychological realm and/or in social and job status (24) but are intimately connected to negative thoughts about oneself that strongly resist suppression in the person’s ego structure (25) and threatening thoughts about the person’s dental treatment with a great impact on the individual´s health (26,27). If individuals also suffer from dental anxiety and/or fear, they may intensify their syndrome profile (28); these disorders can also ap-pear in individuals who are free of mental pathology during procedures such as exodontics, generating a vicious cycle (29,30).

Advancing diagnosis of dental anxiety and/or dental fear is vitally important to controlling and avoiding this vicious cycle if at all possible, as is early psychiatric diag-nosis and completion of scales that examine mental profile and its evolution (31). These measurement instruments are the Hamilton Rating Scale-Anxiety (HRS-A) and the Ham-ilton Rating Scale-Depression (HRS-D), both of which have good psychometric properties and have been validated in Spanish, in 2002 by Lobo *et al*. (31) and in 1988 by Ramos-Brieva *et al*. (32), respectively.

The Clinical application of the data seeks to evaluate whether use of clinical videos to provide information about this surgical intervention is especially dangerous for these patients, as has been found in other studies of populations without psychiatric conditions.

The goal of this study is first to validate the psychometric properties of these two scales, second to evaluate the level of anxiety and depression in survey respondents without mental pathology as compared to the population that suffers from the three above-mentioned mental disorders (anxiety-depressive disorder, adaptive disorder and anxiety disorder) following screening of a video on surgical extraction of a lower third molar at three different times (seven days before screening of the video, after screening, and seven days after screening) and third, as mentioned above, the clinical implication that this projection may have in this type of population, to corroborate whether what the literature states is in agreement with my study. In addition, the study also analyses the influence of a series of sociodemographic factors, including sex and place of residence.

## Material and Methods

-Sample selection and protocol followed

A prospective observational experimental study was performed on a sample of 240 Caucasian subjects ages 18-70. The sample was collected by random sampling from a psychiatry outpatient clinic in the city of Malaga from October 2019 to January 2020. After being told about the study and its anonymity, patients were invited to participate and sign their consent form.

The sample population was divided into four groups according the mental pathology from which the participants suffered. Participants were classified based on a pre-liminary interview performed by the clinic’s psychiatrist (J.A.B.S) using the HRS-D or HRS-A, to avoid any bias on the psychiatrist’s part and classified in: mixed anxiety-depressive order, adaptive disorder, anxiety disorder and population without psychological pathology, with 60 patients in each group. This sample of participants was chosen to facilitate the statistical analysis, based on all the variables considered.

The measurement instruments cited were completed one week before the video screening, immediately after the screening, and one week after the screening to evaluate whether any of the disorders mentioned stood out from the rest and whether sex and/or place of residence influenced the results. The video viewed showed surgical extraction of a partially impacted lower third molar (32). It lasted 2 minutes and 21 seconds and had multimedia information, from incision with scalpel, ostectomy, osteotomy and odontosection to final suturing. The video belongs to three authors of the article who have training in oral surgery: E.B.B, D.T.L and J.L.G.P and readers can view the video on In-ternet at the permalink below. https://youtu.be/YriQxJwUPoY.

-Ethical issues

This study conforms to the Helsinki Declaration, and the protocol was approved by the Research Ethics Committee of the University of Seville on 14 January 2019, with a secure verification code: 76030446e8bf49e55f4b0ecab4b6fc43b4128ffd and verification url.

https://www.juntadeandalucia.es/salud/portaldeetica/xhtml/ayuda/verifica rFir-maDocumento.iface/code/76030446e8bf49e55f4b0ecab4b6fc43b4128ffd.

-Statistical analysis

The statistical analysis used SPSS software version 21.0 to determine reliability and validity of the scales with the Alpha Cronbach coefficient (33) and construct validity of the scale items with the Kaiser-Meyer-Olkin (KMO) index. Exploratory factor analysis of the scales was also performed with Varimax rotation to obtain the rotated component matrix and extract the number of factors in which the components could be grouped and the items belonging to them.

Student’s t-Test and ANOVA (34) were used to evaluate the sociodemographic factors for the two samples and for three or more independent samples, respectively.

With this statistical analysis, there are more data collected: modified dental anxiety scales (MDAS) and dental fear (DFS), as well as the variables: age, medication readjustment and academic background. In future articles, we would like to publish them.

## Results

-Psychometric properties of the two scales: Reliability, construct validity and factor extraction

The measures of reliability for the two scales (HRS-A and HRS-D) at the three times of video viewing showed that all of the scales had optimal values above 0.85. These results suggest that the items from the different questionnaires analysed constitute a useful tool for the research goal, as they show good internal consistency with each other.

To determine construct validity of the two scales for the three video screenings, the items were grouped based on their correlations as one factor. The KMO index enables comparison of the size of the coefficients of correlation observed. If the value is between 0.5 and 0, it is not advisable to continue factor analysis. In this study, all coefficients of correlation were above 0.5, indicating that our matrix is suitable for continuing factor analysis (the values of the four scales were above 0.5). Table 1 presents these results.

**Table 1:**
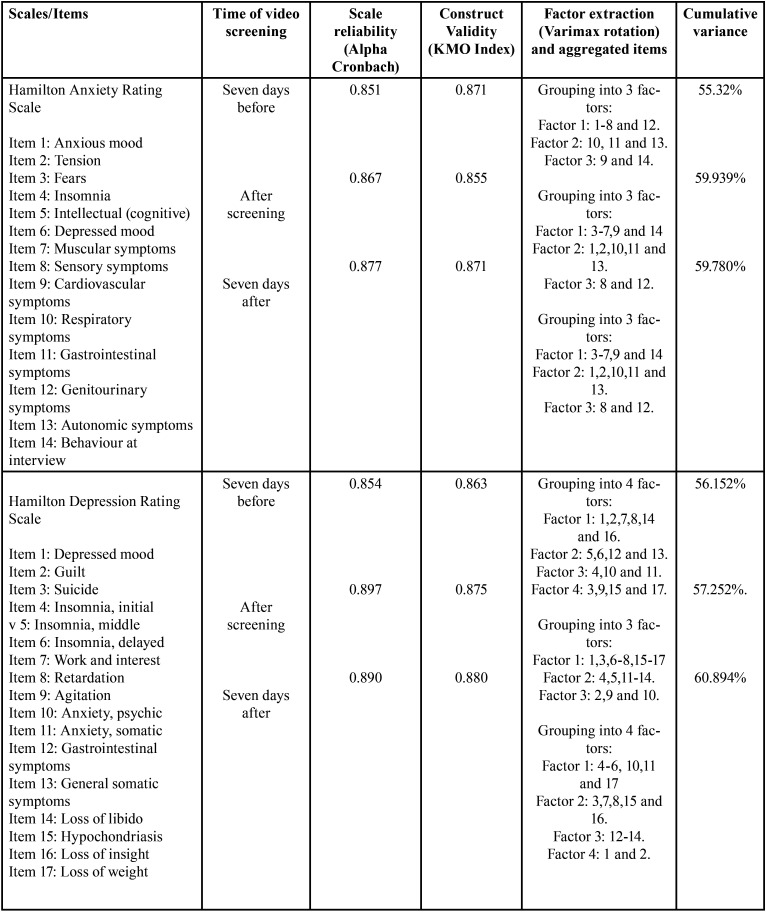
Scale reliability, construct validity and factor extraction at three times of video screening.

In extracting factors from the four scales at the three video screening times, the goal was to find a small number of components that explained the maximum total variance in the original variables. The Varimax method was used to achieve this goal and a rotated component matrix obtained to determine which variables could be included in or discarded from the different factors. Variables with values below 0.5 were discarded. Analyses of the HRS-A and HRS-D, showed that no item need be omitted, as all items had co-efficients greater than or equal to 0.5. Table 2 presents the data.

**Table 2:**
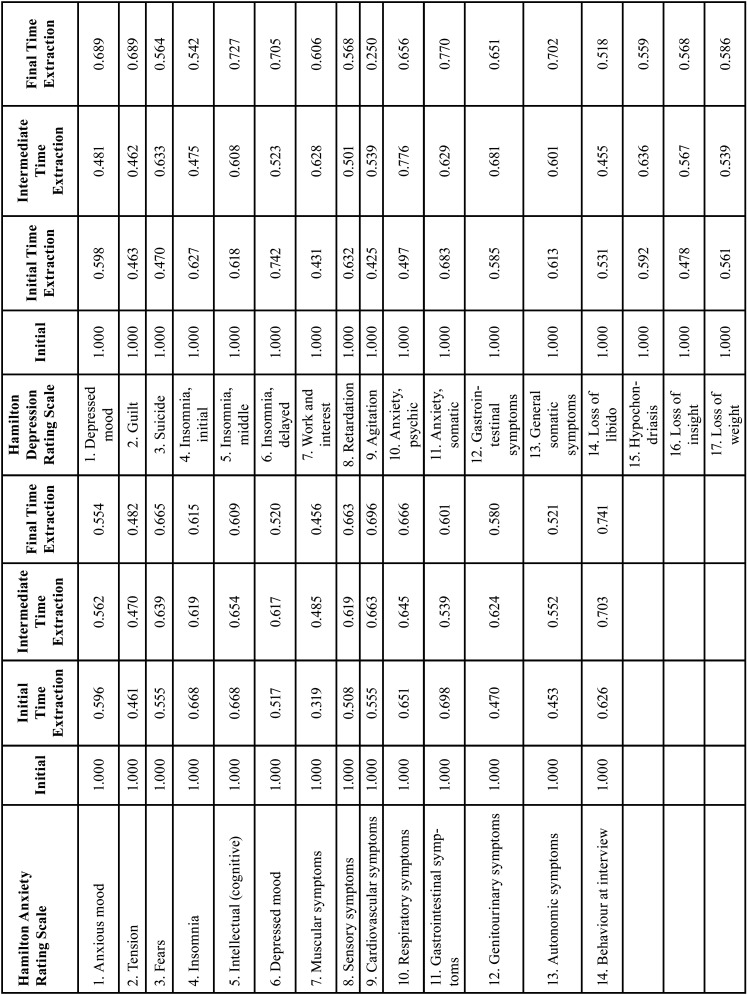
Factor extraction method by item and time of video screening.

-Sociodemographic factors: Sex, place of residence and type of disorder

* Student’s t-Test: The group statistical tests were performed using the test for equality of means based on Student’s t-test and the test for independent samples, accepting that the means are significantly different in the different items when the significance value is below 0.05.

This test was used to analyse sex and place of residence. Tables  Table 3,  Table 3 cont. present the results for sex (Tables  Table 3,  Table 3 cont., Means based on sex, seven days before, after, and seven days after video screening). Tables 4, 4 cont., present the results for place of residence (Tables  Table 4,  Table 4 cont., Means based on place of residence, seven days before, after, and seven days after video screening).

**Table 3:**
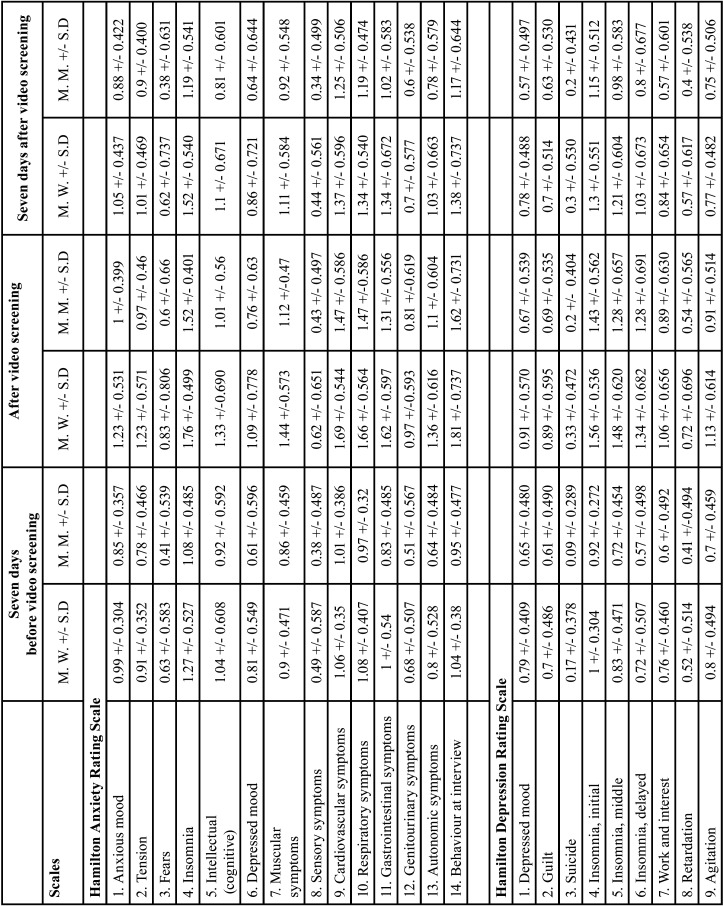
Means based on sex seven days before, after and seven days after video screening.

**Table 3 cont.**
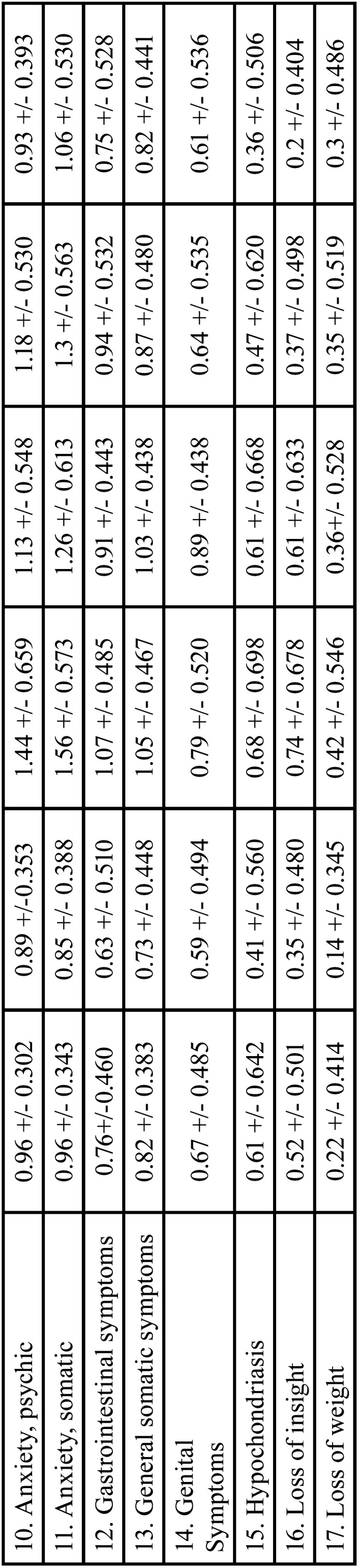
Means based on sex seven days before, after and seven days after video screening.

**Table 4:**
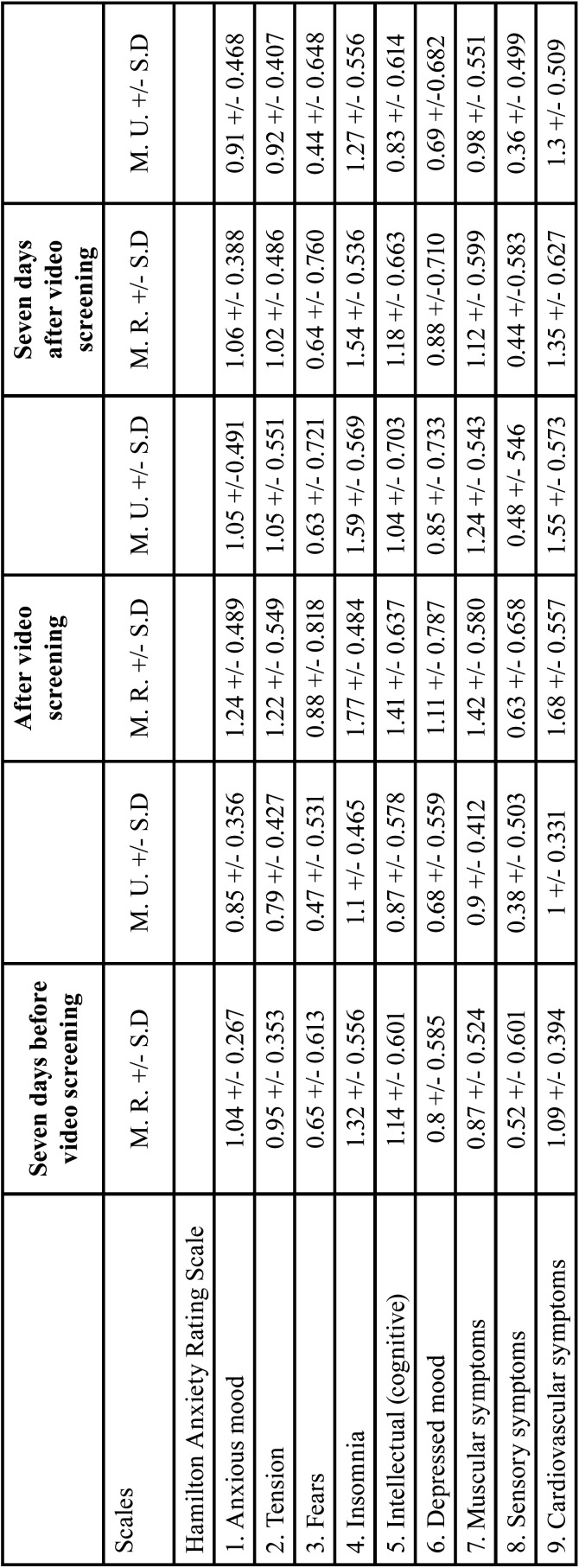
Means based on place of residence seven days before, after and seven days after video screening.

**Table 4 cont.**
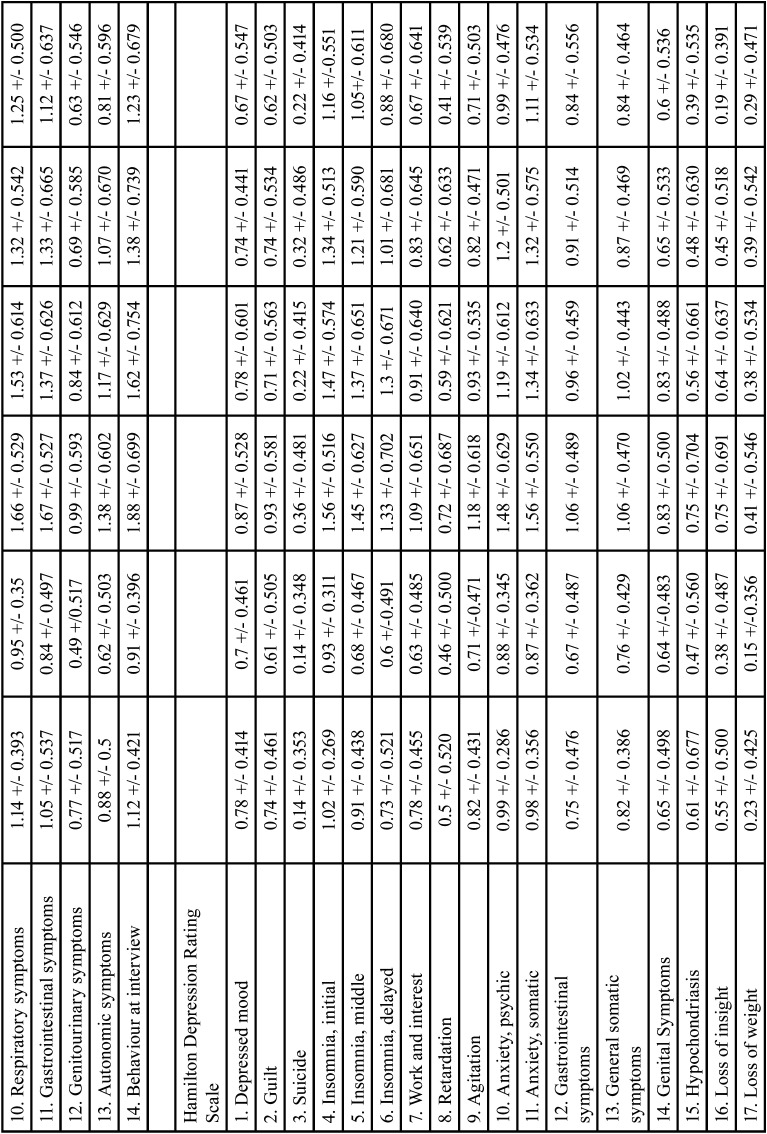
Means based on place of residence seven days before, after and seven days after video screening.

a) Sex: In the analysis by sex, for the time seven days before video screening, the means for women were considerably higher than those for men on all items of all two scales, with the exception of Items 7 (muscular symptoms) and 9 (cardiovascular symptoms) of the HRS-A and items 2 (guilt) and 9 (agitation) of the HRS-D. These items, showed similar values for men and women. After the video screening, the means for women were also higher than those for men on all items on all two scales except items 3 (suicide), 13 (general somatic symptoms) and 17 (loss of weight) of the HRS-D, which was similar to the mean for men. Seven days after video screening, the means for women were higher than those for men on all items on all two scales except item 12 (genitourinary symptoms) of the HRS-A and items 3 (suicide), 11 (anxiety somatic), 13 (general somatic symptoms), 14 (genital symptoms) and 17 (weight loss) of the HRS-D. For these items, women’s means were similar to those for men. None of the items was significant.

b) Place of residence: Analysis by place of residence seven days before video screening showed that the means for rural environment were higher than those for urban environment for all items on all two scales except items 7 (muscular symptoms) of the HRS-A and item 1 (depressed mood), 3 (suicide), 8 (retardation) and 14 (genital symptoms) of the HRS-D. After screening of the video, the means for rural environment were higher than for urban for all items in all two scales except items 6 (delayed insomnia), 13 (general somatic symptoms), 14 (genital symptoms) and 17 (weight loss) of the HRS-D. Seven days after the video screening, the means for rural environment were higher than those for urban for all items of all two scales except items 9 (cardiovascular symptoms) and 12 (genitourinary symptoms) of the HRS-A; and items 13 (general somatic symptoms) and 14 (genital symptoms) of the HRS-D. Since no item was significant, these values were not significant.

* ANOVA: ANOVA of three or more independent samples was used to create the descriptive Tables for Tukey’s Post-Hoc test and Tukey’s HSD test, accepting that the means are significantly different in the different items when the significance value is below 0.05. This test analyses type of disorder, classified as mixed anxiety-depressive disorder, no disorder, adaptive disorder and anxiety disorder. The results are shown in Tables 5,6 and 7 for the three video screenings (Tables  Table 5- Table 7, Means based on type of mental disorder, seven days before, after and seven days after video screening, respectively).

**Table 5:**
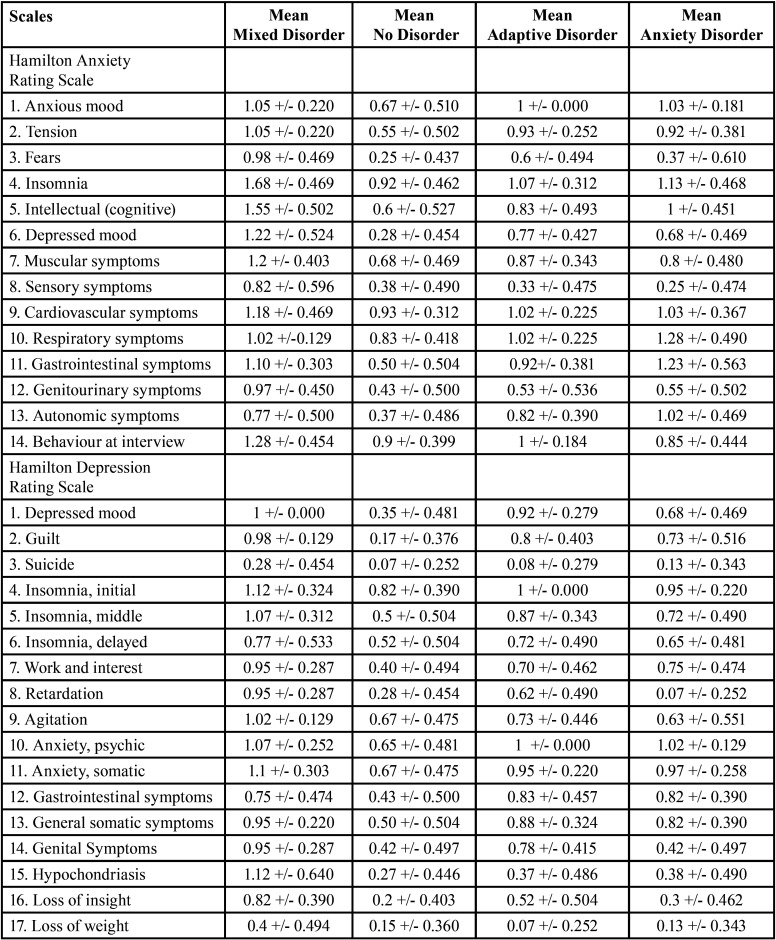
Means based on type of mental disorder seven days before video screening.

**Table 6:**
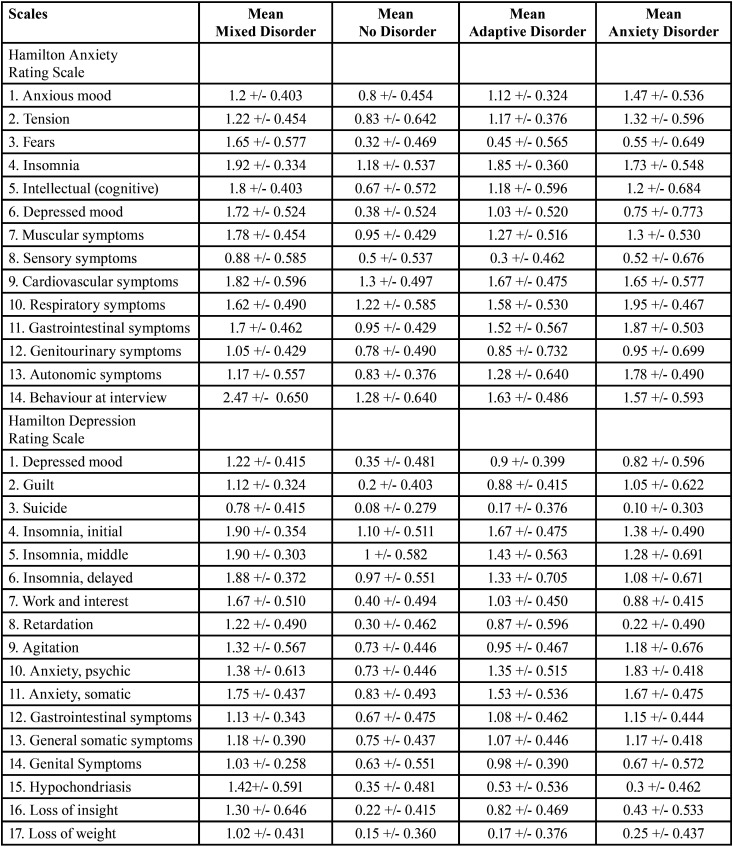
Means based on type of mental disorder after video screening.

**Table 7:**
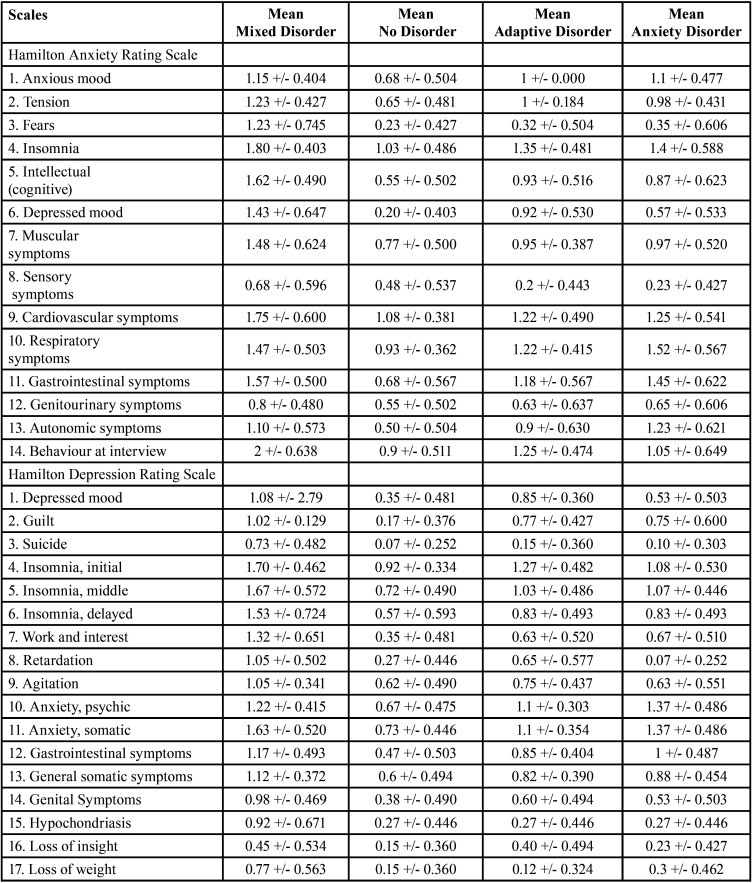
Means based on type of mental disorder seven days after video screening.

a) Type of mental disorder: The results for influence of mental disorder for the three video screening times show that the means are higher seven days before screening in all items of all two scales for the mixed disorder except items 10 (respiratory symptoms), 11 (gastrointestinal symptoms) and 13 (autonomic symptoms) of the HRS-A, where anxiety disorder was higher; and for item 12 (gastrointestinal somatic symptoms) of the HRS-D, where adaptive disorder was higher. After the video screening, the means were higher for mixed disorder for all items on all two scales except items 1 (anxious mood), 2 (tension), 10 (respiratory symptoms), 11 (gastrointestinal symptoms) and 13 (autonomic symptoms) of the HRS-A, where the means for anxiety disorder were higher; and items 10 (anxiety psychic) and 12 (gastrointestinal symptoms) of the HRS-D, where anxiety disorder was higher. Seven days after the video screening, the means were higher for mixed disorder for all items of all two scales except item 13 (autonomic symptoms), of the HRS-A, where the mean for anxiety disorder were higher. Greater psychic imbalance thus occurs in mixed anxiety-depressive disorder, followed by anxiety disorder. No item obtained significantly high means in individuals with adaptive disorder or in the population without mental disorder.

-External validity of the study

External validity denotes whether the results of the study can be extrapolated to a population other than the one used in the study. Depending on eligibility criteria, socio-demographic aspects, age and co-morbidities, applicability of the study and participating center (35).

Based on this definition, our prospective study has 240 Caucasian participants of a wide age range (18 to 70 years), to whom scales (HRS-A and HRS-D) will be administered to determine whether they suffer from any of the mental disorders mentioned above, at three different times during the projection of a video of the third molar. This research was carried out in a psychiatric outpatient clinic in Malaga, this last parameter being the most controversial of all, since it is not a reference center. However, according to the literature reviewed, our results are in accordance with the literature, so our model has worked.

## Discussion

-Psychometric properties of the four scales: Reliability, construct validity and factor extraction

The statistical results for reliability of the scales in this study for all three video screening times, were 0.854, 0.897 and 0.890, respectively, for HRS-D. These values were higher than those described by Ramos Brieva in 1988 (0.72) (32) and by Rehm in 1985 (0.76) (36). For HRS-A at the three screening times, the values were 0.851, 0.867 and 0.877, respectively. These values agree with those obtained in the 2002 study by Lobo *et al*. (0.87) (31).

Construct validity for the three screening times showed KMO values for the HRS-A, recorded were 0.871, 0.55 and 0.871, respectively. These values agree with the 2002 study by Lobo *et al*. (31). In the HRS-D for the three screening times, the KMO values were 0.863, 0.875 and 0.880, respectively. These values cannot be compared to the literature reviewed, however, because the studies by Olden (1986) and Ramos Brieva (1986) use Spearman’s or Pearson’s correlations (32,37).

For factor extraction for the three screening times, 17 items were analysed from the HRS-A. The confirmatory factor analysis indicated that 3 factors had cumulative variance ranging from 55-59%. These results are similar to those obtained by Beneke *et al*. in 1987 but conflict with those obtained by Lobo in 2002 in terms of factor groups. Beneke *et al*. group the items into three factors, whereas Lobo groups them into two. Both studies analysed 17 items (31,38). For the HRS-D, 14 items were analysed. The confirmatory factor analysis identified 4 factors at the first video screening (seven days before) and 3 factors at the intermediate and final times, with cumulative variance of 56-60%. These results disagree with prior studies. In 2009, Olden *et al*. analysed 21 items and grouped them into 5 factors (37) with 42.0% variance. In 1988, Ramos Brieva used a 17-item scale and obtained 4 factors with variance of 56% (32). Ramos Brieva’s results agree with our data on factor grouping. The controversy around the extraction factors may be due to the variety of measurement instruments used in the articles examined, as well as to sample selection and possible mental disorders in the sample.

-Ociodemographic factors: Sex, place of residence and type of disorder

This study’s analysis of sociodemographic factors at the three video screening times concurs with the literature reviewed, although to the extent of our knowledge few studies have been performed on place of residence and type of mental disorder.

As to patients with mental disorders, women experience stronger depressive symptoms or anxiety disorders (39,40) and experience them to a greater extent than do men, in a ratio of 2:1 (41). This prevalence may occur because women are more given to expressing their feelings than are men in socially established archetypes. However, other studies show no differences between sexes in general anxiety or depression, or dental anxiety (24). Patients who come to a dental office tend to have more anxiety about the dental environment (dental chair and dental instruments) and the stimuli related to dental treatment (dental drill and dental injections) (42). In women, both anticipatory anxiety and dental treatment are associated with clinical depression and anxiety, whereas in men they are only related to anticipatory dental anxiety without depression (39).

According to Strine *et al*. (2008), data on behaviour gathered by telephone survey in the US showed that women are more likely to have a life diagnosis of depression and anxiety than are men. Further, a life diagnosis of depression and anxiety is strongly associated with cardiovascular disease, diabetes, anxiety, asthma, obesity and unhealthy behaviour (tobacco, alcohol and physical inactivity) (43). This study found that women scored higher on the HRS-A and HRS-D, at all three video screening times.

As to place of residence, our survey results show that the rural population scored higher than the urban on the HRS-A and HRS-D, at all three video screening times. To our knowledge, this is the only study that analyzes this parameter in these mental disorders and using these scales. However, when compared with patients without mental disorders and after administering DAS (Dental Anxiety Scale), they concluded that the patients who lived in rural areas had a higher level of dental anxiety than those who lived in urban areas (4).

The results for type of disorder show that mixed anxiety-depressive disorder is most likely to disrupt the fragile balance of these patients’ psychic pathology. This population registered greater fluctuation and psychic impact at all three video screening times, results clearly visible in the scores for all items of the scales used. The reason may be the association of the two mental disorders, which are aggravating factors that encourage mental instability (44). Adding dental anxiety and/or fear to anxiety disorder or adaptive disorder can cause the clinical situation to deteriorate, accompanied by worsening of quality of life and oral self-care (28,45), and irregular attendance at dental appointments and/or evasion of them (46). Association of two mental disorders in the same clinical profile—as in mixed disorder—thus provides a much richer symptomatology, with more potential for creating high levels of dental fear than in persons who do not suffer from this pathology. Dental fear has a large endogenous component, leading to greater vulnerability in this type of patient (5,28) and greater impact on quality of life and oral health (47,48).

Among the limitations of our study, the different measurement instruments used in the literature reviewed to assess the different sociodemographic factors analysed, as well as how anxiety and/or depression were tested, hinder development of a uniform criterion.

Very few analyses have been performed to date on patients with any of these three clinical profiles, as some of these mental disorders typically constituted criteria for excluding these populations in prior studies. Further, although both the HRS-A and the HRS–D are constructs with good psychometric properties for evolutionary diagnosis of patients with anxiety and/or depression and involve some complexity, few studies include them; most studies employ other scales that are easier to use.

This study is pioneering in grouping three mental disorders (anxiety disorder, adaptive disorder and mixed anxiety-depressive disorder) and comparing them to a healthy population free of mental illness to evaluate level of anxiety and depression following screening of a video of surgical extraction of a lower third molar at three very different times (seven days before screening, after screening and seven days after screening). Few prior studies of this subject were found, hence, a cautious interpretation of the results of this study, indicating the need for more in-depth research in this field in subsequent trials.

## Conclusions

Finally, we note that the scales chosen for this study (HRS-A and HRS-D) demostrated good psychometric properties with high reliability and construct validity for all three screening times (seven days before screening, after screening, and seven days later).

Completion of all two scales mentioned after all three screening times show that women score predominantly higher means than men, rural environments higher than urban, and persons with mixed disorders (anxiety-depressive) higher than persons with anxiety disorder, persons with adaptive disorder and the population free of mental disorder.

We must emphasize the early detection of mental disorders, since they can worsen the quality of life and thus intensify a vicious circle that can trigger dental anxiety, dental fear and avoidance of dental treatment, among others. This can lead to the need for less conservative treatments and therefore, the obligation to perform more surgical procedures, being more traumatic for the patient. Taking special care in this type of mental patients, as well as in women and in residents of rural areas.
